# FastRNABindR: Fast and Accurate Prediction of Protein-RNA Interface Residues

**DOI:** 10.1371/journal.pone.0158445

**Published:** 2016-07-06

**Authors:** Yasser EL-Manzalawy, Mostafa Abbas, Qutaibah Malluhi, Vasant Honavar

**Affiliations:** 1 College of Information Sciences and Technology, Pennsylvania State University, University Park, PA, United States of America; 2 Systems and Computer Engineering, Al-Azhar University, Cairo, Egypt; 3 KINDI Center for Computing Research, College of Engineering, Qatar University, Duha, Qatar; Indian Institute of Science, INDIA

## Abstract

A wide range of biological processes, including regulation of gene expression, protein synthesis, and replication and assembly of many viruses are mediated by RNA-protein interactions. However, experimental determination of the structures of protein-RNA complexes is expensive and technically challenging. Hence, a number of computational tools have been developed for predicting protein-RNA interfaces. Some of the state-of-the-art protein-RNA interface predictors rely on position-specific scoring matrix (PSSM)-based encoding of the protein sequences. The computational efforts needed for generating PSSMs severely limits the practical utility of protein-RNA interface prediction servers. In this work, we experiment with two approaches, random sampling and sequence similarity reduction, for extracting a representative reference database of protein sequences from more than 50 million protein sequences in UniRef100. Our results suggest that random sampled databases produce better PSSM profiles (in terms of the number of hits used to generate the profile and the distance of the generated profile to the corresponding profile generated using the entire UniRef100 data as well as the accuracy of the machine learning classifier trained using these profiles). Based on our results, we developed FastRNABindR, an improved version of RNABindR for predicting protein-RNA interface residues using PSSM profiles generated using 1% of the UniRef100 sequences sampled uniformly at random. To the best of our knowledge, FastRNABindR is the only protein-RNA interface residue prediction online server that requires generation of PSSM profiles for query sequences and accepts hundreds of protein sequences per submission. Our approach for determining the optimal BLAST database for a protein-RNA interface residue classification task has the potential of substantially speeding up, and hence increasing the practical utility of, other amino acid sequence based predictors of protein-protein and protein-DNA interfaces.

## Introduction

Protein-RNA interactions play key roles in many biological processes including protein synthesis, DNA repair, DNA replication, regulation of gene expression, and viral replication [1–5]. Because of the high cost and the technical difficulties associated with experimentally solving the 3D structure of protein-RNA complexes [6, 7] the number of solved structures represent a small fraction of possible protein-RNA complexes [8]. Hence, several tools have been developed for computational prediction of protein-RNA interfaces [8–10]. These methods are broadly categorized into: i) Structure-based methods (e.g., [11–15]); and Sequence-based methods (e.g., [9, 16–22]). Structure-based methods take as input the (solved or predicted) unbound structure of a query protein whereas sequence-based methods take as input the primary sequence of a query protein. Two recent comparative studies [8, 9] have shown that the state-of the-art sequence-based protein-RNA predictors (e.g., those trained using machine learning methods using position specific scoring matrix (PSSM) based representation of protein sequences) are competitive with their structure-based counterparts. A recent comparative study [23] suggested that the performance of PSSM based methods is better than that of methods based on physio-chemical characteristics of amino acid residues.

PSSM profiles of proteins are generated using the PSI-BLAST program, which is part of the NCBI BLAST package [24]. Given a query amino acid sequence, PSI-BLAST searches the query sequence against a reference database of protein sequences, called BLAST database, to determine homologs of the query sequence (e.g., hits) and uses multiple sequence alignment of the collected hits and the query sequence to generate a PSSM profile. Unfortunately, PSSM profile generation is time consuming and hence limits the practical utility of existing sequence-based methods on large-scale data. In fact, the vast majority of protein-RNA interface prediction methods, implemented as online web servers, limit submissions to only one protein sequence at a time (see for example, BindN+ [19] and PPRInt [20]). One approach to reducing the run time of PSI-BLAST is to use a parallel implementation of NCBI BLAST (e.g., mpiBLAST [25]) which could be executed on high performance computing platforms consisting of tens of thousands of processors. However, not all researchers have access to such high performance computing platforms.

Against this background, we explore an alternative approach to reducing the run time of PSI-BLAST, namely, reducing the size of the BLAST database used to construct the PSSM profiles. In this work, we address the following questions: (i) Given *D*, a BLAST database of protein sequences (e.g., UniRef database [26]), is there a subset of *D* that could be used by PSI-BLAST instead of *D* without an appreciable deterioration in the predictive performance of the resulting protein-RNA interface predictors?; (ii) If so, how can one obtain the smallest possible subset of *D* that meets our criterion?; (iii) How does the decrease in the size of the reference database of sequences used by PSI-BLAST translate into corresponding reductions in the memory and run time needed by PSI-BLAST (and hence, protein-RNA interface predictors that rely on PSI-BLAST)? To the best of our knowledge, this is the first work that systematically studies the pairwise relations between the size of the BLAST database and the performance of PSI-BLAST (in terms of memory usage and run time), the quality of the generated PSSM, and the accuracy of the developed PSSM-based protein-RNA interface predictor (respectively). Based on our results, we developed and implemented FastRNABindR, an improved version of the original RNABindR protein-RNA interface prediction server [9]. FastRNABindR is two orders of magnitude faster than RNABindR without any drop in predictive performance. FastRNABindR has been made available to the scientific community as an online web server accessible at: http://ailab.ist.psu.edu/FastRNABindR/. Unlike RNABindR which limits submission to a maximum of 20 sequences, FastRNABindR accepts up to 500 proteins per submission and returns prediction results within approximately an hour. This research sets the stage for significantly speeding up a broad range of protein sequence classification and sequence labeling tasks that make use of PSSM based representation of the query sequences, including protein-DNA interface residue prediction [27, 28], protein solvent accessibility prediction [29–32], protein dynamics prediction [33, 34], and prediction of vaccine candidates [35, 36] to facilitate high throughput analyses of very large numbers of proteins.

## Materials and Methods

### Data

#### Protein-RNA datasets

For cross-validation experiments, we used the benchmark dataset, RB198 [9]. RB198 dataset was derived from PDB [37] in May 2010 by extracting all protein-RNA complexes in PDB and filtering out complexes that do not meet the following criteria: i) Structures resolution is less than 3.5 Å; ii) The length of the protein chain has to be at least 40 amino acid; iii) The length of the RNA chain has to be at least 5 nucleotides; iv) The number of interface residues in the protein chain has to be at least 3 residues. An amino acid residue was considered an interface if it contains at least one atom within 5 Å of any atom in the bound RNA; v) Protein chain should share at most 30% sequence identity with all other chains in the dataset. The dataset and its identified interfaces are publicly available at: http://ailab1.ist.psu.edu/RNABindR/rb198seq.txt. For running 5-fold cross-validation experiments [38], we split sequences in RB198 dataset into five subsets of almost equal size (see S1 Text). Table 1 lists the number of interfaces and non-interfaces in each RB198 subset.

**Table 1:** Number of interface and non-interface residues in RB198, RB44, and RB111 datasets.

Dataset	No. of Interface residues	No. of Non-interface residues
RB198_1	1666	7618
RB198_2	1636	11456
RB198_3	1496	8805
RB198_4	1452	8365
RB198_5	1700	9466
RB44	1956	4521
RB111	3305	34255

Data for RB198 is provided for each cross-validation fold.

For independent test evaluations, we used the benchmark test set, RB44 [8, 9]. RB44 is a dataset of 44 RNA-binding proteins released between January 1st and April 28th 2011 from the PDB. No two protein chains in this dataset share more than 40% sequence identity [8, 9]. Our analysis of the RB44 using the CD-HIT program [39] shows that RB44 is non-redundant at a sequence similarity threshold of of 30%. RNA-binding residues in protein sequences have been identified using the same cutoff distance used with RB198 dataset. The dataset annotated with the identified interface residues is publicly available at: http://ailab1.ist.psu.edu/RNABindRPlus/rb44.txt.

For comparing our final model, FastRNABindR, with other protein-RNA interface prediction servers, we used the RB111 benchmark dataset [17]. Like RB44, RB111 is also non-redundant at a sequence identity threshold of 30% (using the CD-HIT program [39]). It consists of 111 protein chains extracted from protein-RNA complexes deposited in the PDB between June 2010 to December 2010, and May 2011 to March 2014. The number of interface and non-interface residues in RB44 and RB111 datasets are provided in Table 1.

It is worth noting that although the two independent test sets, RB44 and RB111, are non-redundant at 30% sequence identity, the sequence identity between any test sequence from RB44 or RB111 and the sequences in our training dataset, RB198, is less than 40% [17]. In order to to allow direct comparisons with previous studies [9, 17], we used the same settings as those used in [9, 17].

### Interface residue definition

To the best of our knowledge, there is no gold standard for defining interface residues in a protein-RNA complex. Computational methods reported in the literature for predicting protein-RNA interface residues have used a range of distance cutoffs from 3.5 to 7 Å for determining whether an atom from a protein molecule (and hence an amino acid residue) interacts with an atom from some RNA molecule (and hence a nucleic acid residue) [9]. Following previous studies [9, 13, 16, 17], we used a distance cutoff of 5 Å in defining interface residues. This allows for direct comparisons of our method with RNABindR v2 [9] and previously reported results using RB44 and RB111 datasets [9, 17].

#### UniRef databases

For extracting evolutionary features of protein sequences (i.e., position-specific scoring matrices (PSSMs), we ran PSI-BLAST [24] against several variants of the UniProt Reference Clusters (UniRef) database [26]. First, we downloaded UniRef100 (UR100) and UniRef50 (UR50) as of January 14, 2015. UR100 contains all UniProt Knowledgebase records plus selected UniParc records. In this database, all identical sequences and sub-fragments with 11 or more residues are placed into a single cluster and a representative protein sequence is selected. UniRef90 (UR90) is derived from UR100 using a 90% sequence identity threshold using the CD-HIT algorithm [39]. Similarly, UR50 is derived from UR90 using CD-HIT algorithm and a 90% sequence identity cutoff. We also generated UR40 and UR30 from UR50 and UR40 (respectively) using KClust program [40] and 40% and 30% sequence identity cutoffs. Using a dual octa-core processors machine (Intel Xeon E5-2690) with 128 GB RAM and each processor has a speed of 2.9 GHz and 20 MB cache, KClust took 8 and 15 days to extract UR40 and UR30 sequences (respectively). Finally, we generated six random databases from UR100 (UR50R, UR40R, UR30R, UR10R, UR5R, and UR1R). For the first three random datasets, we randomly extracted sequences from UR100 such that the number of sequences in the random database equals the number of sequences in the corresponding similarity reduced database (i.e., UR50 and UR50R have the same number of sequences). For the last three random databases, URkR (for k = 10, 5, and 1), we randomly extracted k% of UR100 sequences from UR100. Table 2 shows the number of protein sequences in UR100 and its variants.

**Table 2:** Number of protein sequences in UniRef100 database and its variants.

Database	No. of sequences
UR100	50,371,270
UR50	11,992,242
UR50R	11,992,242
UR40	9,893,262
UR40R	9,893,262
UR30	8,888,952
UR30R	8,888,952
UR10R	5,037,127
UR5R	2,518,564
UR1R	503,713

### Distance between two profiles

PSI-BLAST takes as input a query protein sequence and compares it to a protein database, using the gapped BLAST program [41]. The output of PSI-BLAST is simply a 2-D matrix with rows corresponding to residues in the query protein sequence and 20 columns corresponding to the standard 20 amino acids.

Let *Q* denote a query protein of *L* amino acids, *P*1 and *P*2 be two profiles of the query protein *Q* generated by running PSI-BLAST to compare *Q* to databases *D*1 and *D*2, any two BLAST databases considered in our experiments. We can define the distance between proteins *P*1 and *P*2 we use the distance between their respective PSSM profiles [42, 43]. In our study, we used the Normalized Sum of Squared Distance (NSSD) and Normalized Kullback-Leibler (NKL) divergence which are defined as follows:

$$\begin{array}{c} NSSD\left(P1,P2\right)=\frac{1}{20\times L}\overset{}{\underset{}{\Sigma_{i=1}^{L}\Sigma_{j=1}^{20}({\left(P1\left(i,j\right)-P2\left(i,j\right)\right)}^{2}}} \end{array}$$

$$\begin{array}{c} NKL\left(P1,P2\right)=\frac{1}{2\times \left(20L\right)} \end{array}\Sigma_{i=1}^{L}\Sigma_{j=1}^{20}P1\left(i,j\right)log\frac{P1\left(i,j\right)}{P2\left(i,j\right)}+P2\left(i,j\right)log\frac{P2\left(i,j\right)}{P1\left(i,j\right)}$$

### Feature extraction

For each protein sequence in the data set, we generated a PSSM profile by applying PSI-BLAST to carry out three iterations of search (using an *e*-value of 0.001) against the UR100 database. Then, we normalized values in the PSSM matrix using the logistic function. Specifically, each element in the PSSM matrix, *x*, is replaced with $f\left(x\right)=\frac{1}{1+e^{-x}}$. Then, each residue in a given query protein sequence, is encoded using a contiguous window of 25 residues (as done in RNABindR [9]) with the target residue at the center of the window flanked by 12 sequence neighbors to the left and right. We encoded each residue in the sequence window with a 20-element vector extracted from its normalized PSSM profile. Thus, the input to the protein-RNA interface predictor is a target residue encoded by a vector of 25 × 20 = 500 numeric features. The corresponding label (the desired output of the classifier) is 1 if the target residue is an interface residue and 0 otherwise. We experimented with nine alternative representations of the data by repeating the above procedure using nine different variants of UniRef database (e.g., UR50, UR50R, UR40,..etc).

### Performance evaluation

We experimented with three machine learning algorithms that have been widely used for developing biomolecular sequence labeling tools: Naive Bayes (NB) [44]; Random Forest [45] with 100 trees (RF100), which integrates bagging [46] with the random selection of subset feature for training decision trees; and Support Vector Machine [47] with linear (SVML) and radial basis function (SVMRBF) kernels. The three algorithms are implemented as part of the WEKA machine learning workbench [48], which was used in our experiments. We assessed the predictive performance of the classifiers using Accuracy (ACC), Sensitivity (*S*_*n*_), Specificity (*S*_*p*_), and Mathew Correlation Coefficient (*MCC*) measures defined as follows [49, 50]:

$$\begin{array}{c} ACC=\frac{TP+TN}{TP+FP+TN+FN} \end{array}$$ (1)

$$\begin{array}{c} S_{n}=\frac{TP}{TP+FN} \end{array}$$ (2)

$$\begin{array}{c} S_{p}=\frac{TN}{TN+FP} \end{array}$$ (3)

$$\begin{array}{c} MCC=\frac{TP\times TN-FP\times FN}{\sqrt{\left(TN+FN)(TN+FP)(TP+FN)(TP+FP\right)}} \end{array}$$ (4)

where TP, FP, TN, and FN are the numbers of true positive (correctly classified interface residues), false positive(non-interface residues classified as interfaces), true negative(correctly classified non-interface residues), and false negative(interface residues classified as non-interfaces).

The above metrics depend on the classification threshold used to convert predicted class probabilities into binary class labels. In contrast, the Receiver Operating Characteristic (ROC) curve [51] describes the performance of the classifier over all possible thresholds. The ROC curve is a two-dimensional plot in which the true positive rate is plotted on the *Y* axis and the false positive rate is plotted on the *X* axis. Each point on the ROC curve represents the behavior of the classifier at a specific choice of the threshold. The area under ROC curve (AUC) is equivalent to the probability that a randomly chosen positive example will be ranked higher than a randomly chosen negative example. Any AUC score higher than 0.5 is considered better than random guessing. The ideal classifier will have an AUC equals 1. In the Results section, we limit our discussion to the AUC and report other threshold-dependent metrics in the Supporting Information (S2 Text).

We assessed the performance of the PSI-BLAST program by recording the total running time taken to generate PSSM profiles for a given dataset (e.g., RB198 and RB44) and the maximum amount of memory used during the entire execution period for a given dataset. Time and memory measurements are taken using the Linux utility commands, time and top. All profile generation experiments (as well as sequence similarity reduction using KClust [40]) were conducted using a single processor on a dual octa-core processors machine (Intel Xeon E5-2690) with 128 GB RAM. Each processor has 2.9 GHz clock speed and 20 MB cache.

## Results and Discussions

### PSSM profile generation limits the applicability of existing methods

Table 3 summarizes the existing protein-RNA interface residue prediction methods that meet the following criteria: i) the method is available in the form of an online web server; ii) the method uses PSI-BLAST to generate PSSM profiles for submitted query protein(s). Out of the 7 servers listed, only 3 allow batch submission (i.e., submission of more than a single query protein). RBScore [52] accepts up to 5 query sequences while RNABindR v2 [9] and RNABindRPlus [17] accept up to 20 query sequences. The available documentation for many of these servers acknowledge that the computational requirements of PSI-BLAST search impact the usability of the servers. Servers often limit the number of query sequences allowed per user over a specified timeframe or disallow batch submissions that contain more than a single query protein at a time. For instance, BindN+ server [19], which limits the submission to one sequence, states in its submission page that “Because of the PSI-BLAST search, BindN+ runs more slowly than BindN. Please be patient”. Table 3 also shows that 6 out of 7 methods run PSI-BLAST against databases of more than 50 million protein sequences. In the remainder of this Section, we empirically show that that the use of extremely large BLAST databases has severe implications for the computational requirements of PSI-BLAST (in terms of run time and memory usage) without commensurate improvements in the predictive performance of the classifiers built using the resulting PSSM profiles.

**Table 3:** List of existing Protein-RNA interface residue prediction servers that requires generation of PSSM profiles for query sequence(s).

Method	BLAST database	BLAST database size	No. of sequences	URL
BindN+	UniProtKB	50371270	1	http://bioinfo.ggc.org/bindn+/
PPRInt	NCBI nr	78002046	1	http://www.imtech.res.in/raghava/pprint/
PRBR	NCBI nr	78002046	1	http://www.cbi.seu.edu.cn/PRBR/
RBScore	Swiss-Prot	462,819	≤5	http://ahsoka.u-strasbg.fr/rbscore/
RNABindR v2.0	NCBI nr	78002046	≤20	http://ailab1.ist.psu.edu/RNABindR/
RNABindRPlus	NCBI nr	78002046	≤20	http://ailab1.ist.psu.edu/RNABindRPlus/
SNBRFinder	NCBI nr	78002046	1	http://ibi.hzau.edu.cn/SNBRFinder/

BLAST database size refers to the size of the database as of February 2016 and not the precise size of the database used by the servers. No. of sequences refers to the maximum number of protein sequences that can be processed by the corresponding server in a single submission.

### More data is not always better

Table 4 shows the AUC of four classifiers estimated using 5-fold cross-validation on ten different PSSM based representations of RB198 dataset generated using UR100 and its variants. It is striking that none of the four classifiers achieves its best AUC (estimated using cross-validation) when the classifiers are trained using the PSSM representation obtained by running PSI-BLAST against the largest database, UR100. The same conclusion holds when the four classifiers are trained using RB198 and tested using RB44 test set (see Table 5).

**Table 4:** Performance comparison using cross-validation tests.

Features	NB	RF100	SVML	SVMRBF
UR100	0.75	0.75	0.77	0.79
UR50	0.73	0.77	0.79	0.80
UR50R	0.73	0.76	0.78	0.80
UR40	0.70	0.77	0.78	0.80
UR40R	0.73	0.76	0.78	0.80
UR30	0.70	0.76	0.78	0.80
UR30R	0.73	0.76	0.78	0.80
UR10R	0.76	0.77	0.78	0.80
UR5R	0.75	0.77	0.78	0.80
UR1R	0.74	0.77	0.78	0.79

AUC of different classifiers using 5-fold cross-validation and 10 different variants of PSSM based encodings generated using UR100 database and its variants.

**Table 5:** Performance comparison using independent tests.

Features	NB	RF100	SVML	SVMRBF
UR100	0.69	0.72	0.77	0.78
UR50	0.74	0.78	0.78	0.80
UR50R	0.70	0.76	0.79	0.80
UR40	0.73	0.77	0.78	0.80
UR40R	0.71	0.76	0.78	0.80
UR30	0.73	0.78	0.79	0.80
UR30R	0.72	0.77	0.79	0.80
UR10R	0.78	0.80	0.79	0.81
UR5R	0.76	0.78	0.79	0.81
UR1R	0.75	0.78	0.78	0.79

AUC of different classifiers trained using RB198 and tested using RB44 for 10 different variants of PSSM based encodings generated using UR100 database and its variants.

### What is an optimal UniRef database?

In light of the results presented in the previous section, it is natural to ask whether we can identify an optimal UniRef database, i.e., the one with the smallest number of protein sequences, and hence the fastest time for running PSI-BLAST and computing PSSMs that could be used to develop a classifier with the best predictive performance. Results in Table 4 suggest that there is no single database that is optimal across all the classifiers. The AUC for the NB ranges from 0.70 to 0.76 and the best AUC is reached when the database UR10R is used to generate the PSSM profiles. RF100 has AUC values in the range 0.75–0.77 and the best AUC is observed using 5 variants of UniRef database (the smallest database, UR1R, is one of them). SVML has AUC values in the range 0.77–0.79 and the best performance is achieved using UR50 database. Finally, SVMRBF has AUC scores between 0.79 and 0.80 and the best performance is observed using 8 out of the 10 UniRef databases (UR5R is the smallest database that leads to the best AUC). However, if we consider both the cross-validation results (Table 4) and independent test results (Table 5), we can identify a single database that appears to be optimal across all the classifiers. The best performance of all classifiers using RB44 test set is reported using UR10R. On the cross-validation experiments, all classifiers (except SVML) have the highest AUC reported using UR10R database. On the other hand, the best performance of SVMRBF observed using UR10R on both cross-validation and independent test evaluations is also reported using UR5R. Next, we show how different database size reduction approaches affect the performance of PSI-BLAST and the quality of the generated PSSM profiles.

### Similarity reduced versus random sampled databases

So far, we have shown that using UR100 database for extracting proteins PSSM profiles does not provide classifiers with the best predictive performance in terms of AUC estimated using both cross-validation and independent test experiments and there exist subsets of UR100 database that lead to improvements in classifiers performance. In this section, we address two interesting research questions: i) What is the best way to generate subsets of UR100?; ii) How does the decrease in the database size affect the computation performance of PSI-BLAST (in terms of computation time and memory)? To address the first question, we generated subsets of UniRef database (see Methods section) using two approaches: i) Standard tools for reducing sequence similarity; ii) Random sampling. To address the second question, we ran all PSI-BLAST experiments on a dedicated single machine (single run at a time) and recorded the time taken by the PSI-BLAST run (in hours), the maximum used memory (in gigabytes) for each run.

**Fig 1:**
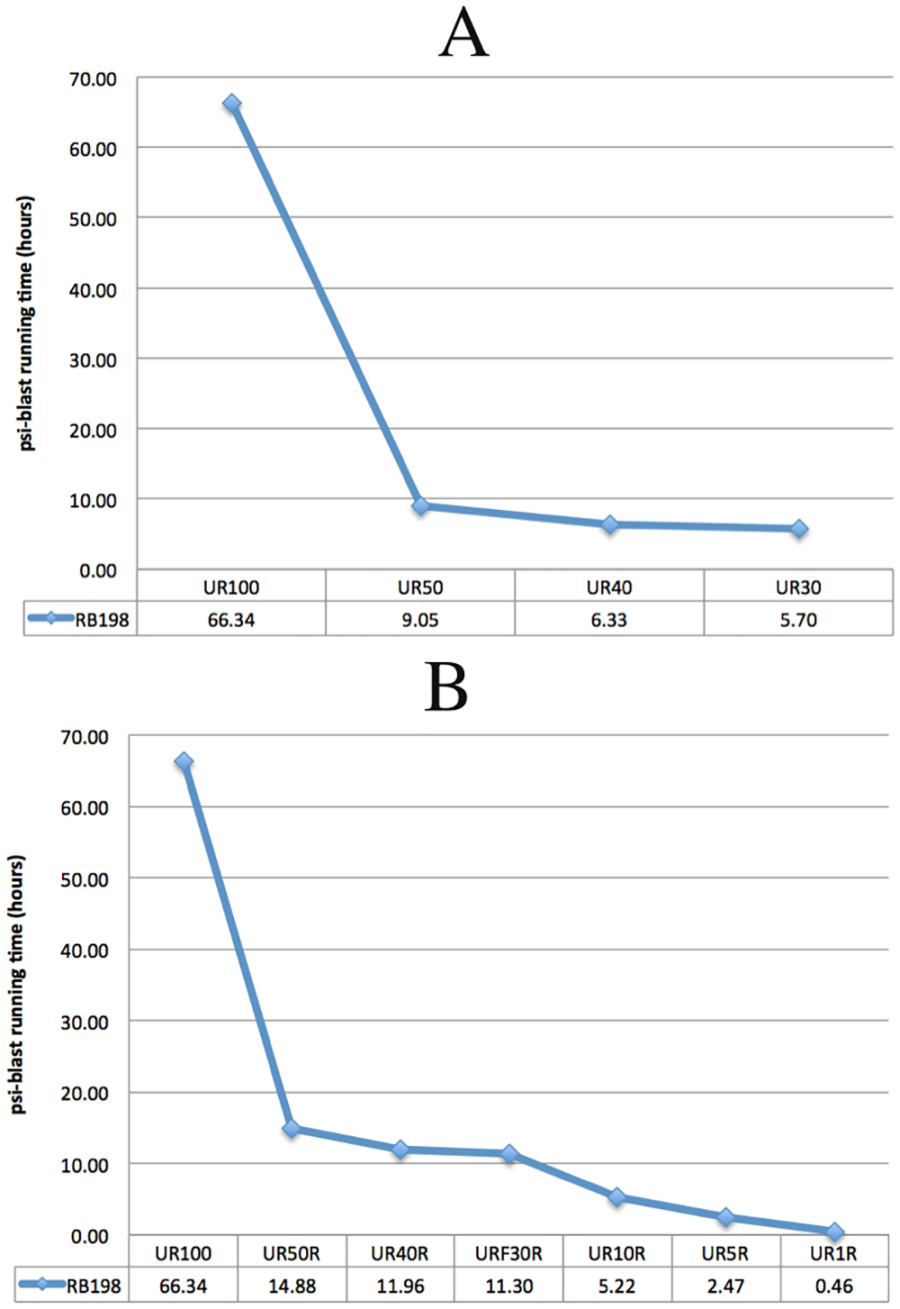
PSI-BLAST run time. The total PSI-BLAST run time (in hours) for generating PSSM profiles for RB198 sequences using UniRef100 versus its sequence similarity reduced variants (A) and its random sampled variants (B).

Fig 1A shows a monotonic decrease in PSI-BLAST run time used to generate PSSM profiles for sequences in RB198 dataset when searching against UniRef databases with different sequence identity cutoffs (UR100, UR50, UR40, and UR30). Fig 1B shows a similar pattern when searching against UR100 and randomly sampled variants (UR50R, UR40R, UR30R, UR10R, UR5R, and UR1R). Interestingly, the PSI-BLAST run time drops from 66.34 hours to 5.22, 2.47, and 0.46 hours, when UR10R, UR5R, and UR1R (respectively) are used as the reference database for PSI-BLAST runs.

**Fig 2:**
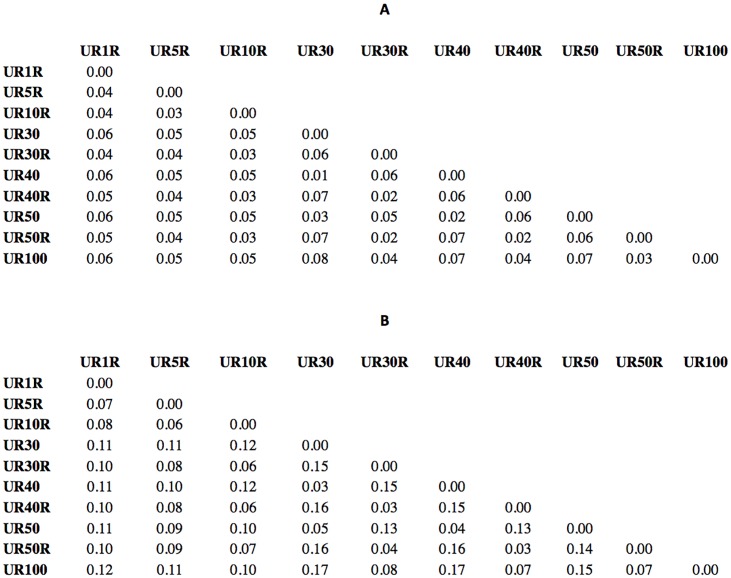
Average pairwise distances between different PSSM profiles of RB198 sequences. Average pairwise NSSD (A) and NKL (B) distances over RB198 PSSM profiles. Random sampled UniRef variants are more representatives of UR100 than similarity reduced UniRef variants.

Another interesting observation from Fig 1 is that PSI-BLAST run time using UniRef similarity reduced databases (UR50, UR40, and UR30) is better than that using randomly sampled UniRef databases with the same number of sequences (UR50R, UR40R, and UR30R). Table 6 shows another difference between similarity reduced UniRef variants and random sampled UniRef variants. Similarity reduced UniRef variants consume less memory than their corresponding random sampled UniRef variants. In addition, Table 7 shows that the number of hits used to build the PSSM profiles using random sampled UniRef variants is higher than those returned when using similarity corresponding reduced UniRef variants. These observations collectively suggest that random sampled databases are more representative than the similarity reduced databases of the same size (in terms of the number of sequences). In fact, Fig 2 shows that, using RB198 dataset, the average pairwise distances between PSSMs generated using UR100 and random sampled UniRef databases is smaller than the average pairwise distances between PSSMs generated using UR100 and similarity reduced UniRef databases.

**Table 6:** PSI-BLAST memory usage.

Database	RB198	RB44
UR100	12.00	12.00
UR50	3.50	3.50
UR50R	4.20	4.20
UR40	2.80	2.70
UR40R	3.50	3.50
UR30	2.40	2.40
URF30R	3.10	3.10
UR10R	1.80	1.80
UR5R	0.91	0.89
UR1R	0.21	0.20

Maximum computation memory (in gigabytes) allocated for PSI-BLAST during the generation of PSSMs profiles for RB198 and RB44 datasets using UniRef100 and its variants.

**Table 7:** Average number of hits used for generating PSSM profiles.

Features	RB198	RB44
UR100	453	492
UR50	362	331
UR50R	422	433
UR40	318	261
UR40R	415	416
UR30	295	239
URF30R	413	416
UR10R	393	371
UR5R	336	291
UR1R	166	99

Average number of hits found by PSI-BLAST when generating PSSMs profiles for RB198 and RB44 datasets using UniRef100 and its variants.

In summary, we have shown that the run time as well as the maximum memory used monotonically decrease with the decrease in the size of the reference database used by PSI- BLAST. We also showed that reference databases obtained by randomly sampling UniRef data yield larger number of hits for constructing PSSM profiles, and hence yield more representative PSSM profiles than those obtained from similarity reduced UniRef databases of the same size.

### FastRNABindR method and web server

The results summarized in the preceding section set the stage for implementing a protein-RNA interface prediction web server that can process large numbers of query sequences and return predictions in a reasonable amount of time. Our experimental results (See Tables 4 and 5 and Fig 1) suggest that we should use the SVMRBF classifier trained using UR5R PSSM profile representation to implement FastRNAbindR because this classifier (i) has the highest AUC on both cross-validation data and independent test data and (ii) yields more than one order of magnitude reduction in PSI-BLAST run time for generating PSSM profiles (from 66.3 hours to 2.5 hours for 198 sequences). Also, the amount of memory needed by PSI-BLAST is decreased from 12 GB to 0.89 GB (Note that we could use UR100 with restricted amount of memory (i.e., less than 12 GB) but this might increase the run time). Compared with RNABindR v2.0 server [9], this is a significant improvement in computation time. RNABindR v2.0 takes 10–15 minutes per sequence while our recommended classifier takes less than one minute per sequence. Also, the RNABindR v2.0 server which also implements a classifier trained using RB198 dataset, has a reported AUC of 0.82 on the RB44 test set [9], whereas our recommended classifier has an AUC of 0.81 on the RB44 test set.

Our results show that switching from UR5R to UR1R database would reduce the PSI-BLAST run time for generating PSSM profiles for the 198 protein chain sequences in RB198 from 2.5 to less than 0.5 hours but the AUC of the SVMRBF classifier would drop from 0.81 to 0.79 when evaluated using RB44 test set. It is interesting to explore if we could further reduce the run time of our server, by using UR1R instead of UR5R, without sacrificing the predictive performance. To achieve this goal, we used UR1R to generate the PSSM profiles and replaced the single SVMRBF classifier with consensus classifier that returns the average of predicted probabilities from SVMRBF and RF100 classifiers. The consensus classifier, which has the advantage of reduced run time for PSI-BLAST, has an AUC of 0.81 when tested using RB44 dataset. An online web server, FastRNABindR, for fast prediction of protein-RNA interfaces using the consensus classifier is freely accessible at: http://ailab.ist.psu.edu/FastRNABindR/. In addition to the web server, a stand-alone version of FastRNABindR has been made freely available to the scientific community. The stand-alone version is hardware and operating system independent since it is implemented in Java. However, to run FastRNABindR on one’s own machine, two third party freely available programs need to be installed: WEKA machine learning workbench [48]; and NCBI BLAST+ [24].

### Comparison with existing protein-RNA interface prediction servers

Table 8 reports the results of comparing FastRNABindR with 3 protein-RNA interface prediction servers that utilize PSSM profiles for representing interface and non-interface residues in amino acid sequence (RNABindR v2 [9], BindN+ [19], and PPRInt [20]) and 2 structure-based protein-RNA interface prediction servers (KYG [11] and PRIP [13]) using RB111 as an independent test set. Interestingly, FastRNABindR outperforms RNABindR v2 based on 3 out of the 4 reported metrics of performance. We notice that no single method outperforms all other methods using the four observed metrics of performance. Due to data imbalance (RB111 data has 34255 non-interface residues and 3305 interface residues), higher accuracy might be associated with predictors that have low sensitivity (e.g., low true positive rate). In this case, MCC often provides a more balanced evaluation of performance than ACC [49]. Among the 6 prediction servers, FastRNABindR and BindN+ have the highest MCC of 0.24. However, due to the long run time of PSI-BLAST search against the extremely large NCBI nr database used by BindN+, BindN+ server limits user submission to only one sequence per submission. On the other hand, FastRNABindR server accepts up to 500 sequences per submission.

**Table 8:** Evaluation of servers using RB111 test set.

Method	ACC (%)	*S*_*n*_	*S*_*p*_	MCC
FastRNABindR	75.1	0.61	0.76	0.24
RNABindR v2	72.0	0.63	0.73	0.22
BindN+	83.5	0.43	0.87	0.24
PPRInt	76.1	0.48	0.79	0.18
KYG	77.5	0.47	0.80	0.19
PRIP	75.2	0.45	0.78	0.15

Results in Table 8 should be viewed as comparisons between different protein-RNA interface prediction servers. Such comparisons are interesting from users’ perspectives and for understanding the strengths and weakness of different tools. The predictors reported in Table 8 have been developed using different training data and different design decisions (e.g., distance cutoff for defining interface residues) have been made by the developers of these tools. Therefore, it is inappropriate to interpret these results as direct comparisons between the underlying methods for these servers. Fair and direct methods comparisons require unified experimental settings, which is satisfied only for comparing RNABindR v2 and FastRNABindR.

## Conclusions

Ever since the advent of the first biomolecular sequence databases in the 1980s, homology search has become one of the most common and important tasks in bioinformatics. The sequence databases used for homology search (i.e., NCBI BLAST databases) are regularly updated to improve their coverage. Currently, NCBI nr BLAST database has more than 78 million protein sequences and this number is expected to further increase as ongoing sequencing projects generate additional data. The generation of PSSM profiles is an important application of homology search and PSSM encoding of protein sequences is a widely used feature representation for developing protein functional site predictors. Due to the large size of the BLAST databases, generating PSSM profiles is a computational bottleneck for many bioinformatics tools. In this work, we experimented with two approaches to reduce the size of the BLAST database, random sampling and similarity reduction, and showed that random sampled databases provide better PSSM profiles in terms of number of hits used to generate the profile and the distance between that profile and the corresponding one generated using the full BLAST database. Based on our findings, we developed and implemented FastRNABindR, a tool for accurate and fast prediction of protein-RNA interface residues. FastRNABindR uses only 1% of UniRef100 data to generate PSSM profiles. With this substantial reduction in the size of the BLAST database, we report more than 100-fold improvement in computation time while the predictive performance is better than that obtained using the entire UniRef100 data or at least as good as the best performance observed using eight more variants of UniRef100 considered in our experiments.

In this work, we assessed the quality of the PSSM profiles generated using PSI-BLAST search against UR100 database and its variants using three performance metrics: PSI-BLAST run time; PSI-BLAST memory usage; and the predictive performance of the resulting protein-RNA interface predictor developed using PSSM profiles as input features. It would be interesting to analyze the protein sequences (PSI-BLAST hits) used to generate the PSSM profiles. Such analysis might help inform the development of methods to improve the quality of the PSSM profiles to be used as input features for protein-RNA interface predictors. Work in progress is aimed at: i) Exploring more sophisticated approaches (e.g., based on clustering analysis of protein sequences) to determine the optimal BLAST database for a given classification task; ii) Applying the proposed methodology to develop reliable yet computationally efficient methods for related amino acid sequence labeling (e.g., protein-DNA interface residue prediction) and sequence classification (e.g., identifying RNA-binding proteins). (iii) Exploring whether there is a single optimal BLAST database that can be used across multiple tasks (e.g., protein-RNA, protein-DNA, and protein-protein interface prediction) or whether the optimal BLAST database is task-dependent; iv) Developing parallel or distributed implementations and/or advanced data structures to further reduce the run time and memory usage of the methods in order to support very high throughput analyses.

## Supporting Information

S1 Text Partitioning of RB198 data into five subsets for cross-validation experiments. (DOCX)

S2 Text Threshold-dependent metrics of classifiers performance estimated using cross-validation and independent tests. (DOCX)
