# Supplementary material for: FastRNABindR: Fast and Accurate Prediction of Protein-RNA Interface Residues
Source: PLoS One. 2016 Jul 6;11(7):e0158445. doi: 10.1371/journal.pone.0158445 (PMC4934694; doi:10.1371/journal.pone.0158445)
Supplement: S1 Text — (DOCX) [file pone.0158445.s001.docx]

Partitioning of RB198 sequences into five subsets of almost equal number of amino acid sequences for 5-fold cross-validation experiments.

Set_1

1SI3_A

3HUW_V

3GIB_A

3L25_A

3EPH_A

1R3E_A

2ZI0_A

1GTF_L

2VQE_P

1ASY_A

2VQE_L

3I5X_A

3CIY_A

2VQE_K

1PGL_2

1WPU_A

1M8V_A

1E8O_A

2J01_5

2VQE_N

1VQO_Q

2CZJ_A

2VQE_I

1VQO_W

1VQO_P

2VQE_Q

2QBE_6

3A6P_H

1VQO_Y

1KNZ_A

1VQO_X

2PJP_A

1B23_P

2AZ0_A

2OZB_B

3IEV_A

1FFY_A

2QUX_A

1VQO_B

2IY5_B

Set_2

2DER_A

1C0A_A

1T0K_B

2CT8_A

1VQO_C

1DDL_B

1VFG_A

1J2B_A

2ANR_A

2VQE_F

3HTX_A

2BH2_A

3FHT_A

1WZ2_A

3HL2_A

2VQE_R

2R7R_A

3IAB_A

3K62_A

1VQO_A

1J1U_A

2VQE_O

3I1M_F

1VQO_3

2VQE_S

1JBS_A

2J01_V

1FEU_A

2FK6_A

2JLV_A

3KIQ_Y

1VQO_D

2DU3_A

1H3E_A

1R9F_A

1HR0_W

1SDS_A

2Q66_A

1UN6_B

1N78_A

Set_3

2VQE_B

2BGG_A

3EX7_A

2GTT_A

1QF6_A

1VQO_T

2AZX_A

1VQO_E

1VQO_K

2GXB_B

1DFU_P

2WJ8_A

1OOA_A

1A34_A

2PO1_A

2F8S_A

2I82_A

3EGZ_A

1VQO_V

3ICQ_T

3EQT_A

2J01_R

1M8X_A

3FOZ_A

1VQO_I

2GJW_A

2VQE_H

2J01_T

2VQE_E

2J01_6

2J01_I

2J01_3

2J01_P

1VQO_S

2ZUE_A

2HW8_A

2J01_7

2D6F_C

1VQO_2

1VQO_M

Set_4

1VQO_N

1AV6_A

2ZJR_R

3I1M_U

2VQE_G

3HAX_C

2J01_Q

1H4S_A

2NQP_A

2VQE_D

3D2S_A

2NUG_A

2VQE_T

1VQO_Z

1N35_A

1Q2R_A

2J01_1

1DI2_A

1F7U_A

2BX2_L

2A8V_A

2VOO_A

2J01_0

1HQ1_A

2GIC_A

1VQO_H

1VQO_U

2W2H_A

2J01_N

2E9T_A

3F1E_X

3DD2_H

2Z2Q_E

1WSU_A

2ZZM_A

1TFW_A

1MZP_A

2VQE_J

1VQO_R

Set_5

1VQO_J

2GJE_D

3I1N_D

2BTE_A

1U0B_B

3IAB_B

2J01_F

1FXL_A

2VNU_D

2J01_W

1ZBH_A

2J01_U

1QTQ_A

1VQO_1

3BSO_A

2VQE_M

1JID_A

1LNG_A

2B3J_A

1YVP_A

2F8K_A

2J01_8

1VQO_L

1UVJ_A

2ZNI_A

2ZKO_A

2VQE_C

1SER_A

2IPY_A

2J01_4

2R8S_H

2BU1_A

2FMT_A

1K8W_A

2GJE_A

3BT7_A

2J01_H

2ASB_A

2IY5_A
