# Supplementary material for: FastRNABindR: Fast and Accurate Prediction of Protein-RNA Interface Residues
Source: PLoS One. 2016 Jul 6;11(7):e0158445. doi: 10.1371/journal.pone.0158445 (PMC4934694; doi:10.1371/journal.pone.0158445)
Supplement: S2 Text — (DOCX) [file pone.0158445.s002.docx]

**Supplementary Table 1**. Accuracy (ACC) of different classifiers estimated using 5-fold cross-validation on RB198 and 10 different variants of PSSM based encodings generated using UR100 database and its variants.

| Features | NB | RF100 | SVML | SVMRBF |
| --- | --- | --- | --- | --- |
| UR100 | 0.699 | 0.682 | 0.867 | 0.725 |
| UR50 | 0.732 | 0.763 | 0.778 | 0.729 |
| UR50R | 0.703 | 0.702 | 0.719 | 0.728 |
| UR40 | 0.628 | 0.766 | 0.721 | 0.727 |
| UR40R | 0.675 | 0.708 | 0.717 | 0.727 |
| UR30 | 0.620 | 0.763 | 0.719 | 0.724 |
| UR30R | 0.680 | 0.709 | 0.720 | 0.731 |
| UR10R | 0.703 | 0.741 | 0.723 | 0.731 |
| UR5R | 0.696 | 0.752 | 0.719 | 0.728 |
| UR1R | 0.671 | 0.742 | 0.710 | 0.718 |

**Supplementary Table 2**. Sensitivity (S*_n_*) of different classifiers estimated using 5-fold cross validation on RB198 and 10 different variants of PSSM based encodings generated using UR100 database and its variants.

| Features | NB | RF100 | SVML | SVMRBF |
| --- | --- | --- | --- | --- |
| UR100 | 0.668 | 0.698 | 0.215 | 0.713 |
| UR50 | 0.578 | 0.610 | 0.523 | 0.715 |
| UR50R | 0.613 | 0.679 | 0.702 | 0.720 |
| UR40 | 0.663 | 0.596 | 0.686 | 0.710 |
| UR40R | 0.653 | 0.665 | 0.696 | 0.716 |
| UR30 | 0.664 | 0.587 | 0.681 | 0.707 |
| UR30R | 0.661 | 0.668 | 0.703 | 0.719 |
| UR10R | 0.660 | 0.647 | 0.699 | 0.721 |
| UR5R | 0.672 | 0.637 | 0.694 | 0.715 |
| UR1R | 0.682 | 0.638 | 0.686 | 0.702 |

**Supplementary Table 3**. Specificity (S*_p_*) of different classifiers estimated using 5-fold cross validation on RB198 and 10 different variants of PSSM based encodings generated using UR100 database and its variants.

| Features | NB | RF100 | SVML | SVMRBF |
| --- | --- | --- | --- | --- |
| UR100 | 0.703 | 0.679 | 0.981 | 0.726 |
| UR50 | 0.757 | 0.789 | 0.824 | 0.730 |
| UR50R | 0.718 | 0.706 | 0.721 | 0.728 |
| UR40 | 0.620 | 0.795 | 0.726 | 0.728 |
| UR40R | 0.677 | 0.715 | 0.720 | 0.728 |
| UR30 | 0.611 | 0.793 | 0.725 | 0.726 |
| UR30R | 0.681 | 0.715 | 0.722 | 0.732 |
| UR10R | 0.708 | 0.756 | 0.727 | 0.731 |
| UR5R | 0.697 | 0.770 | 0.722 | 0.729 |
| UR1R | 0.668 | 0.760 | 0.713 | 0.720 |

**Supplementary Table 4**. Matthew's correlation coefficient (MCC) of different classifiers estimated using 5-fold cross validation for RB198 and 10 different variants of PSSM based encodings generated using UR100 database and its variants.

| Features | NB | RF100 | SVML | SVMRBF |
| --- | --- | --- | --- | --- |
| UR100 | 0.280 | 0.278 | 0.329 | 0.332 |
| UR50 | 0.266 | 0.321 | 0.336 | 0.338 |
| UR50R | 0.254 | 0.288 | 0.319 | 0.340 |
| UR40 | 0.207 | 0.318 | 0.311 | 0.332 |
| UR40R | 0.247 | 0.287 | 0.314 | 0.335 |
| UR30 | 0.201 | 0.309 | 0.307 | 0.327 |
| UR30R | 0.256 | 0.289 | 0.320 | 0.341 |
| UR10R | 0.278 | 0.314 | 0.321 | 0.342 |
| UR5R | 0.278 | 0.321 | 0.315 | 0.336 |
| UR1R | 0.258 | 0.310 | 0.299 | 0.317 |

**Supplementary Table 5**. Accuracy (ACC) comparisons of different classifiers trained using RB198 and tested using RB44 for 10 different variants of PSSM based encodings generated using UR100 database and its variants.

| Features | NB | RF100 | SVML | SVMRBF |
| --- | --- | --- | --- | --- |
| UR100 | 0.598 | 0.643 | 0.658 | 0.656 |
| UR50 | 0.639 | 0.696 | 0.659 | 0.665 |
| UR50R | 0.645 | 0.690 | 0.667 | 0.676 |
| UR40 | 0.585 | 0.690 | 0.674 | 0.671 |
| UR40R | 0.638 | 0.686 | 0.662 | 0.673 |
| UR30 | 0.567 | 0.689 | 0.663 | 0.668 |
| UR30R | 0.645 | 0.692 | 0.669 | 0.677 |
| UR10R | 0.671 | 0.702 | 0.669 | 0.677 |
| UR5R | 0.625 | 0.682 | 0.669 | 0.675 |
| UR1R | 0.583 | 0.677 | 0.656 | 0.665 |

**Supplementary Table 6**. Sensitivity (S*_n_*) of different classifiers trained using RB198 and tested using RB44 for 10 different variants of PSSM based encodings generated using UR100 database and its variants.

| Features | NB | RF100 | SVML | SVMRBF |
| --- | --- | --- | --- | --- |
| UR100 | 0.708 | 0.703 | 0.789 | 0.791 |
| UR50 | 0.769 | 0.736 | 0.785 | 0.824 |
| UR50R | 0.681 | 0.695 | 0.800 | 0.821 |
| UR40 | 0.812 | 0.732 | 0.791 | 0.827 |
| UR40R | 0.681 | 0.700 | 0.797 | 0.820 |
| UR30 | 0.828 | 0.733 | 0.797 | 0.838 |
| UR30R | 0.715 | 0.699 | 0.811 | 0.820 |
| UR10R | 0.782 | 0.747 | 0.810 | 0.835 |
| UR5R | 0.823 | 0.744 | 0.803 | 0.834 |
| UR1R | 0.853 | 0.742 | 0.801 | 0.823 |

**Supplementary Table 7**. Specificity (S*_p_*) of different classifiers trained using RB198 and tested using RB44 for 10 different variants of PSSM based encodings generated using UR100 database and its variants.

| Features | NB | RF100 | SVML | SVMRBF |
| --- | --- | --- | --- | --- |
| UR100 | 0.550 | 0.616 | 0.602 | 0.597 |
| UR50 | 0.583 | 0.679 | 0.604 | 0.596 |
| UR50R | 0.630 | 0.689 | 0.609 | 0.614 |
| UR40 | 0.488 | 0.671 | 0.624 | 0.603 |
| UR40R | 0.619 | 0.679 | 0.604 | 0.609 |
| UR30 | 0.453 | 0.669 | 0.605 | 0.595 |
| UR30R | 0.614 | 0.688 | 0.608 | 0.615 |
| UR10R | 0.623 | 0.682 | 0.609 | 0.608 |
| UR5R | 0.540 | 0.655 | 0.611 | 0.606 |
| UR1R | 0.467 | 0.650 | 0.594 | 0.597 |

**Supplementary Table 8**. Matthew's correlation coefficient (MCC) of different classifiers trained using RB198 and tested using RB44 for 10 different variants of PSSM based encodings generated using UR100 database and its variants.

| Features | NB | RF100 | SVML | SVMRBF |
| --- | --- | --- | --- | --- |
| UR100 | 0.237 | 0.294 | 0.359 | 0.357 |
| UR50 | 0.324 | 0.383 | 0.357 | 0.386 |
| UR50R | 0.286 | 0.356 | 0.375 | 0.399 |
| UR40 | 0.281 | 0.372 | 0.381 | 0.395 |
| UR40R | 0.276 | 0.351 | 0.369 | 0.394 |
| UR30 | 0.268 | 0.371 | 0.369 | 0.399 |
| UR30R | 0.303 | 0.360 | 0.385 | 0.399 |
| UR10R | 0.372 | 0.396 | 0.385 | 0.408 |
| UR5R | 0.337 | 0.367 | 0.381 | 0.405 |
| UR1R | 0.304 | 0.360 | 0.363 | 0.387 |
